# The Medical Bulletin Visiting List or Physician's Call Record

**Published:** 1891-01

**Authors:** 


THE MEDICAL BULLETIN VISITING LIST OR PHYSICIAN'S CALL RECORD.—Arranged upon an original and convenient monthly and weekly plan for the daily recording of professional visits. F. A. Davis, Publisher, Phila., 1891.

The Visiting List contains a Calendar for the last six months of 1890, all of 1891 and 1892; Table of Signs to be used in Keeping Accounts; Table of Fees, Dr. Ely's Obstetrical Table; Tables for Calculating the Number of Doses in a given R, etc, etc.; for Converting Apothecaries' Weights and Measures into Grammes; Metrical Avoirdupois and Apothecaries' Weights; Number of Drops in a Fluidrachm; Graduated

Doses for Children; Graduated Table for Administering Laudanum; Periods of Eruption of the Teeth; The Average Frequency of the Pulse at Different Ages in Health; Formulæ and Doses of Hypodermic Medication; Use of the Hypodermic Syringe; Formulæ and Doses of Medicines for Inhalation; Formulæ for Suppositories for the Rectum; The Use of the Thermometer in Disease; Poisons and their Antidotes; Treatment of Asphyxia; Anti-Emetic Remedies; Nasal Douches; Eye washes, etc., etc. It is published in three styles according to the number of pages and blanks for recording visits.

